# Evaluation and characterization of tumor lysis syndrome before and after chemotherapy among pediatric oncology patients in Tikur Anbessa specialized hospital, Addis Ababa, Ethiopia

**DOI:** 10.1186/s12878-018-0117-0

**Published:** 2018-09-04

**Authors:** Haileleul Micho, Yasin Mohammed, Daniel Hailu, Solomon Genet

**Affiliations:** 1 Department of Biomedical Sciences, College of Health Sciences, Dilla University, Dilla, Ethiopia; 2 Department of Medical Biochemistry, School of Medicine, College of Health sciences, Addis Ababa University, P. O. Box 9086, Addis Ababa, Ethiopia; 3 Department of Pediatrics and child Health, School of Medicine, College of Health Sciences, Addis Ababa University, Addis Ababa, Ethiopia

**Keywords:** Tumor lysis syndrome, Pediatric oncology, Cell death, Biomarkers

## Abstract

**Background:**

Tumor lysis syndrome (TLS) is a life-threatening emergency disorder, caused by an abrupt release of intracellular metabolites after tumor cell death. It is characterized by a series of metabolic manifestations, especially hyperuricemia, hyperkalemia, hyperphosphatemia and hypocalcemia. The aim of this study was to evaluate and characterize the incidence of tumor lysis syndrome among pediatric oncology patients before and after treatment.

**Methods:**

Hospital based prospective cohort study was conducted for 6 months on 61 newly diagnosed pediatric oncology patients. Socio-demographic data was collected by interview administered questionnaire. Patients were followed and the physical diagnosis, imaging and laboratory results were interpreted by senior physicians. Data was entered to and analyzed by SPSS version 23.

**Results:**

Among 61 pediatric oncology patients 39(63.9%) were males. The mean (±SD) age of the pediatric patients was 6.39 (± 3.67) years ranging from 2 months to 14 years. 29.5% of patients were found to have TLS. There were 11.5% and 18.0% of laboratory TLS (LTLS) and clinical TLS (CTLS) cases respectively. There were72.2% spontaneous and 27.8% treatment induced TLS cases with 23% and 21.3% cases of hyperuricemia and 4.9% and 6.6% cases of hyperkalemia incidence before and after treatment respectively. Only two patients died, in the study period, due to TLS.

**Conclusion:**

There was high incidence of TLS irrespective of socio-demographic variation among study participants, suggesting that children with cancer are at risk of developing TLS. As TLS is a life-threatening complication of malignancies, early identification of patients at risk and reducing morbidity and mortality is crucially important.

## Background

Tumor lysis syndrome (TLS) is one of the most common cancer therapy related complication, first described by Bedrna and Polcák in 1929 [1]. It is a life-threatening condition with high morbidity and mortality, caused by an abrupt release of intracellular metabolites after tumor cell lysis [2]. It usually occurs a few hours to a few days after commencing cytotoxic chemotherapy for tumors with a high percentage of proliferating and drug-sensitive cells. Cell death leads to the release of potassium, phosphate, uric acid, proteins and other purine metabolites into the systemic circulation. These factors will overtax the body’s homeostatic mechanisms and overwhelm the capacity of the renal system for normal excretion of these materials. When the renal clearance of these chemical substances is overwhelmed, hyperkalemia, hyperuricemia, hyperphosphatemia and secondary hypocalcemia will result. Serum lactate dehydrogenase (LDH) levels are also often elevated concurrently [3]. Uncontrolled TLS progresses to lactic acidosis and acute renal failure (ARF). Clinically, this results in multi organ defects such as acute kidney injury (AKI), cardiac arrhythmias, and seizures or sudden death that requires intensive care. TLS is the most common oncologic emergency, and without prompt recognition and early therapeutic intervention, morbidity and mortality is high [4]. Hande and Garrow first initiated a definition of the clinical and pathologic characteristics of patients at risk for developing TLS. They classified TLS as laboratory TLS (LTLS) or clinical TLS (CTLS) [5]. Cairo and Bishop modified these criteria to formulate a commonly used classification system for TLS. This system defines LTLS when two or more of the following abnormalities are met within 3 days before or 7 days after the initiation of chemotherapy in the face of adequate hydration and use of uric acid lowering agent: 25% decrease from baseline in serum calcium, and/or 25% increase from baseline in the serum values of uric acid, potassium, or phosphate.

CTLS is defined as LTLS accompanied by one or more clinical manifestations such as cardiac arrhythmia, AKI, seizure, or death with an elevated serum creatinine > 1.5ULN (upper limit of normal) [6]. TLS is the mainstay for the complication and unfavorable outcome of cancer treatments. Specifically cancers with high proliferation and high sensitivity to chemotherapy are highly susceptible to TLS and its complications. In the era of novel agents, there is greater concern that the incidence of TLS in pediatric oncology may occur more frequently. The problem is disturbing and causing a major concern in low income countries like Ethiopia. Unfortunately, due to socio-economic and other factors patients come to health facilities at an advanced stage. To the best of our knowledge, the incidence of TLS has not been specifically addressed in Ethiopia. Therefore, determining the frequency, clinical course and outcome of TLS in pediatric oncology patients in our setup will significantly be important to help shape clinical as well as public health care of the patients and the population. Findings from this work can help TLS diagnosis and improve treatment strategies of malignancies. In addition, the results obtained from this study are expected to pave the way for further broad and extensive related studies. In this study we have tried to give baseline information about TLS incidence and treatment outcome, with special emphasis on measurement of laboratory parameters that predict the onset and progress of TLS in different pediatric oncology, like acute myeloid leukemia (AML), chronic myeloid leukemia (CML), acute lymphoid leukemia (ALL), non-Hodgkin lymphoma (NHL), Burkitt’s lymphoma (BL) and some solid tumors.

## Methods

A prospective cohort study was conducted from October, 2016 - July, 2017 at the pediatric hematology/oncology unit of Tikur Anbesa Specialized Hospital (TASH), which is the largest referral teaching hospital of the country, located in Addis Ababa, Ethiopia. The hospital has about 800 beds and gives diagnostic and treatment services for about 370,000–400,000 patients per year. The pediatric hematology/oncology unit of TASH is under the department of pediatrics and child health and gives out patient and in patient services. Around 500–600 pediatric oncology patients visit TASH annually. The unit has hemato-oncologists, hemato-pathologysts, residents, and nurses.

### Study population and data collection

The study population for this study was all newly admitted pediatric patients with hematologic and solid malignancy during the study period in TASH. The data was collected by using structured questionnaires which has been translated into the local language “Amharic”.

### Blood sample collection, processing and laboratory analysis

Venous blood sample was collected using two tubes. About 2 ml of blood in EDTA tube was used for total WBC analysis using Sysmex 2000*i* hematology analyzer (Sysmex, Japan). Blood (5 ml) was drawn into serum separator tube (SST) for clinical chemistry tests (uric acid, creatinine, BUN and LDH) using Mindray clinical chemistry analyzer (Shenzhen Mindray Biomedical Electronics, China) and for electrolyte analysis (potassium, sodium, chloride and calcium). This was done before and after chemotherapy; and duration differs based on stage of malignancy. Prophylactic management was done for all admitted patients.

### Management of TLS

A prophylactic management with intensive hydration, 3000 ml/m^2^/24 h, and a uric acid lowering agent (allopurinol) along with their respective treatments were administered to the patients. Hydration was used to increase urine output via increased kidney clearance.

### Statistical analysis

Data was checked, cleaned, coded and entered to SPSS version 23 for analysis. Simple descriptive statistics was used to present the socio-demographic and clinical characteristics of the study subjects. Data distribution was checked by using Kolmogrov-Smirnov and Shapiro Wilk test. Paired sample *t* test was used for parametric data and wilcoxon signed rank test for the nonparametric data. Data was described by the use of means, standard deviation, median, inter quartile range (IQR) and percentage. While chi-square test was used to compare categorical variables, Pearson and Spearman rho correlation was applied for continuous variables. *p*-value < 0.05 at 95% confidence interval was considered as statistically significant in all the analysis.

## Result

This study enrolled 61 pediatric oncology patients, 39(63.9%) of the study subjects were males. The mean (±SD) age of the pediatric patients was 6.39 (± 3.67) years ranging from 2 months to 14 years. Male to female ratio was 1.77:1. Twenty eight (45.9%) of pediatric patients were found within the age group of 5 to 9 years. Around 95.1% of the study participants were in low income strata. Regarding distribution, 26 (42.6%) of the children were from Oromia region, followed by Southern Nation Nationality Peoples Region 12(19.7%). Forty four (72.1%) of the study participants were from the rural area of the country. Fifty five (90.2%) of the patients, arrived late at the hospital i.e., 7 days from the onset of sign and symptoms of TLS. None of the patients reported history of cancer in their family (Table 1).

**Table 1:** Socio-demographic characteristics of pediatric oncology patients

Variables	Frequency	Percent (%)
Sex		
Male	39	63.9
Female	22	36.1
Age		
0–4	19	31.1
5–9	28	45.9
10–14	14	23.0
Region		
Addis Ababa	8	13.1
Oromia	26	42.6
Amhara	10	16.4
SNNPR	12	19.7
Others ^b^	5	8.2
Residence		
Urban	17	27.9
Rural	44	72.1
Time of arrival in the hospital after the onset of sign and symptom of disease		
Within < 72 h	2	3.3
72 h – 7 days	4	6.6
After 7 days	55	90.2
History of cancer in the family		
Yes	0	0.0
No	61	100.0
Total	100	100.0

^a^, the sixth, seventh, ninth and tenth babies of the families are labeled as “others”

^b^, Tigray and Somali regions are labeled as “others”

**Fig. 1:**
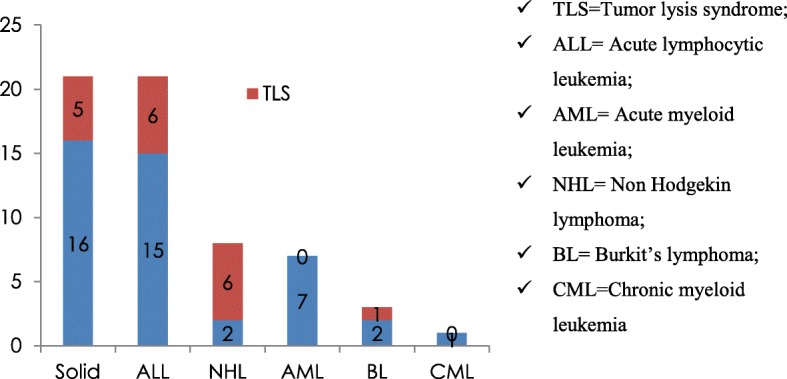
Frequency of TLS among various hematological and solid malignancies of pediatric patients

Out of 61 pediatric oncology patients 40(65.9%) had hematologic malignancy, while the rest 21(34.4%) had solid malignancy. Majority of the hematologic malignancies in our study were ALL, 34.4% (*n* = 21) followed by NHL, 13.1% (*n* = 8), AML, 11.5% (*n* = 7), BL, 4.9% (*n* = 3) and CML, 1.6% (*n* = 1). Types of malignancies and their corresponding magnitude of TLS are shown in Fig. 1.

**Fig. 2:**
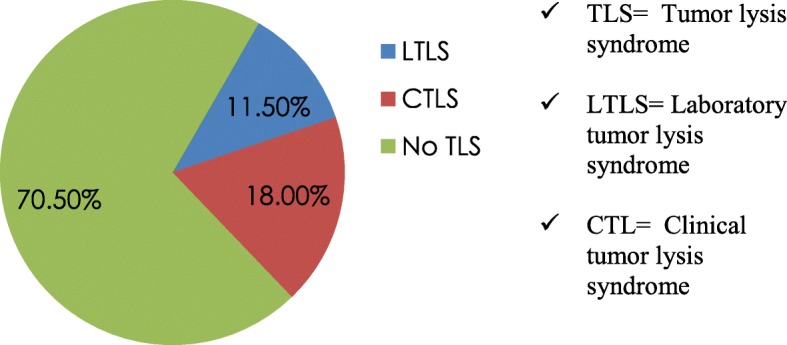
The incidence of LTLS and CTLS in pediatric oncology patients

A total of 18 (29.5%) patients had TLS. Seven (11.5%) cases had LTLS whereas the rest 11 (18.0%) had CTLS (Fig. 2).

Out of the 18 TLS cases, 13 (72.2%) developed spontaneous TLS while the remaining 5 (27.8%) had treatment induced TLS. Sixteen (88.9%) of the TLS cases in the study resolved while 2 (11.1%) died. Around 44.4% of the patients resolved from TLS within 72 h of management with adequate hydration and uric acid lowering agent (allopurinol). There were two death reports (11.1%) considered to be caused by TLS, during the study period (Table 2).

**Table 2:** Management and treatment outcome of TLS in pediatric oncology patients (*n* = 18)

Variables	Frequency	Percent (%)
Resolution		
Yes	16	88.9
No	2	11.1
Total	18	100
Timing of resolution		
Within 72 h	8	44.4
Within 72 – 120 h	5	27.8
Above 120 h	3	16.7
Death	2	11.1

Acute kidney injury was observed in 9 (14.8%) of patients, while cardiac arrhythmia and seizure were observed in single patient each. Easy fatigability, abdominal pain and prolonged fever were the most prevalent complaints by the study participants. There was also a high prevalence of swelling on different parts of the body (jaw, facial, neck, thigh and lower leg) of the study participants. Some of the patients also complained of having headache, cough and bleeding (nasal and gum).

Out of 44 patients who underwent chest x-ray imaging, 33 were normal, 4 showed pleural effusion, 4 had lung opacity, 2 had cardiomegally and 1 showed solitary calcified nodule. Ultra sound and physical diagnosis results showed that 23 (37.7%) and 20 (32.8%) patients had hepatomegally and splenomegaly respectively, 6 (9.8%) had pleural effusion, 33 (54.1%) had lymph node enlargement at different sites of their body and 9 (14.8%) had edema. In addition palmar and conjuctival pallor were observed in 47 (77%) of the study participants.

The mean ± SD levels of biochemical parameters before and after treatment are shown in Table 3. A Wilcoxon signed rank test showed statistically significant decrease in total WBC count and serum levels of LDH and calcium after treatment (*p* < 0.05). The study also showed that there is a statistically significant increase in the serum levels of uric acid, BUN and creatinine after treatment (*p* < 0.05). There is no statistically significant difference (*p* > 0.05) in serum levels of sodium and chloride before and after treatment in the study participants. A two tailed paired sample *t* test revealed that there is a difference between potassium level before treatment (x̅=4.03, SD = 0.74) and after treatment (x̅=4.40, SD = 0.50) in the study participants, and this is statistically significant, t(60) = − 4.37, *p* < 0.0001.

**Table 3:** Laboratory values of biochemical parameters before and after treatment in pediatric oncology patients

Parameters	Before treatment Median(IQR)	After treatment Median(IQR)	*p* value
Potassium(mmol/L)^a^	4.03 ± 0.74	4.40 ± 0.50	**0.0001**
WBC(×10^3^/μl)	12.50(8.400–37.700)	10.00(6.250–18.825)	**0.0001**
LDH(IU/L)	749.00(556.95–1274.00)	524.00(371.5–863.5)	**0.0001**
Uric acid(mg/dl)	4.94(3.15–6.90)	6.20(4.69–6.90)	**0.0001**
BUN(mg/dl)	17.00(12.50–25.50)	19.00(13.00–28.25)	**0.006**
Creatinine(mg/dl)	0.70(0.60–0.80)	0.89(0.80–1.00)	**0.0001**
Sodium (mmol/l)	139.0(137.0–140.9)	139.0(138.0–141.0)	0.719
Calcium (mmol/l)	2.01(1.23–2.30)	1.21(1.09–1.90)	**0.0001**
Chloride (mmol/l)	105.0(101.20–109.00)	105.0(100.1–108.0)	0.671

^a^Potassium is normally distributed, expressed as mean ± standard deviation, the rest of the variables are not normally distributed, expressed as median and IQR

The incidence of hyperuricemia in this study was found to be 23% before treatment and 21.3% after treatment and the incidence of hyperkalemia was 4.9% and 6.6% before and after treatment respectively. The study also revealed two cases of hypocalcemia both before and after treatment.

Bivariate Pearson correlation analysis showed that age was weakly and negatively associated with serum potassium level both before (*r* = − 0.043, *p* = 0.742) and after (*r* = − 0.020, *p* = 0.877) treatment in the pediatric oncology patients, and this is not statistically significant. Spearman’s rho correlation analysis was also used to see correlation between age and some of the dependent variables both before and after treatment in the study participants (Table 4).

**Table 4:** Spearman’s rho correlation between age and some of the dependent variables before and after treatment in pediatric oncology patients

Variable	WBC^a^	WBC^b^	LDH^a^	LDH^b^	UA^a^	UA^b^	BUN^a^	BUN^b^	Cr^a^	Cr^b^	Ca^a^	Ca^b^
**r** AGE **p**	-.05 -.08 -.07 -.06 -.11 -.04 .14 .20 .27^c^.15 -.15 -.14											
	.68 .55 .61 .66 .38 .75 .29 .13 .04 .27 .25 .28											

^a^ before treatment, ^b^ after treatment; *UA* uric acid, *Cr* creatinine, *Ca* calcium

^c^ Correlation is significant at the 0.05 level (2-tailed)

Chi-square (x^2^) test was implemented to check for the association between the categorical independent variables (gender, residence and family income) and the outcome variable (TLS). The analysis showed that none of the independent variables, gender (x^2^ = 1.378, *p* = 0.240), residence (x^2^ = 0.105, *p* = 0.746) and family income (x^2^ = 1.625, *p* = 0.202) showed significant association with the occurrence of TLS in the study participants (Table 5**)**.

**Table 5:** Chi-square test and cross tabulation between independent variables (gender, residence and family income) and TLS in pediatric oncology patients. (Birr is the Ethiopian currency)

Variables	None TLS, No (%)	TLS, No (%)	X^2^	*p*-Value
Gender				
male	30(49.2)	9(14.8)	1.378	0.204
female	13(21.3)	9(14.8)		
Residence				
urban	13(21.3)	4(6.6)	0.105	0.746
rural	30(49.2)	14(22.9)		
Family income				
< 1228 Birr/month/individual)	33(54.1)	17(27.9)	1.625	0.202
> 1228 Birr/month/individual	10(16.4)	1(1.6)		

## Discussion

The incidence of TLS was 29.5%. Though there is a wide variation in TLS occurrence across the world, this result is comparable with previous researches that reported 26.5% and 30.7% incidence of TLS respectively [7, 8]. The LTLS and CTLS incidences were 11.5% and 18.0%; similar to 11.1% LTLS and 19.6% CTLS reported from a cohort study by Darmon et al. [8] but differs from a report of lower incidence of CTLS (2.9%) in Iran and 6.7% in Saudi Arabia [9, 10]. Out of 18 TLS cases, 13 (72.2%) had spontaneous TLS while the remaining 5 (27.8%) developed treatment induced TLS. This is not in agreement with a previous report of 21.9% spontaneous TLS versus 78.1% therapy induced TLS in Saudi Arabia [10] and 20% spontaneous versus 80% therapy induced TLS elsewhere [11]. The difference in incidence rates reported can be attributed to several factors, such as application of slightly different criteria to recognize TLS, difference in study population, age, underlying malignancy, late presentation of the patients to the health facility, stage of disease at the time of diagnosis and the type of anti-cancer drugs used in our facility. For instance highly myelo-suppressive chemotherapies such as high dose methotrexate and cytarabine are used less frequently in our setup. The employment of prophylactic management (i.e., hydration and allopurinol) for all admitted patients may also attribute for a relatively less incidence of chemo induced TLS. But this needs further study to accurately decide on the causes of this discordance.

This study revealed that there were statistically significant decrease in total WBC count and serum levels of LDH after treatment (*p* < 0.05). As the main target of cancer treatment is to eliminate or reduce cancerous cells and a cancer load is mainly manifested by total WBC count (in hematologic malignancies) and serum LDH level, a decrease of these parameters during treatment sounds good outcome of treatment as suggested by Mirrakhimov *et al.* [12]. The decrease in the serum [LDH] after treatment may be due to a decrease in the tumor load. There was statistically significant decrease in the serum level of calcium after treatment (*p* < 0.05). This is because, cytotoxic therapy kills tumor cells leading to rapid release of intracellular phosphate from malignant cells and cancer cells may contain as much as four times the amount of organic and inorganic phosphorous as compared to normal cells [13]. When phosphate concentration is raised, it may lead to precipitation of calcium phosphate crystals, resulting in lowered serum calcium level.

We observed an increase in the serum levels of uric acid, potassium, BUN and creatinine after treatment (*p* < 0.05). As cancer treatment is intended to eliminate or reduce cancerous cells, and when the cells are killed, their intracellular contents will leak out. Thus, there will be an increase in the concentration of these intracellular components and their metabolites in the serum. Uric acid is the end product of purine breakdown. When uric acid is in excess in the serum it results in hyperuricemia. Uric acid may prevent recovery from AKI in TLS, as it has been shown to inhibit proximal tubule cell proliferation [14]. The incidence of hyperuricemia in this study was 23% before treatment and 21.3% after treatment. The result shows that the patients have already developed hyperuricemia before treatment which is in agreement with a result reported from a retrospective study in Turkey which showed a hyperuricemia of 26.5% [6]. But our result is not in agreement with a 40% hyperuricemia, reported from a descriptive study conducted at Liaquat [11]. This variation in the incidence of hyperuricemia may be due to the variation in the types of cancers under study and their response to treatment. It might also be due to late presentation of the patients to the clinics, treatment protocol and difference in study design. Allopurinol was used to reduce or inhibit the formation of uric acid but it has no effect on the already formed uric acid which would have been managed by using a recombinant urate oxidase (rasburicase), but it was not used because it was not available in our set up. The 4.9% and 6.6% incidence of hyperkalemia in the study participants before and after treatment respectively was comparable with a research report from Iran, of 2.9% and 5.8% incidence of hyperkalemia before and after treatment respectively [9]; but discordant with another research which reported a 23% incidence of hyperkalemia and 12% hypokalemia [10]. There was no hypokalemia case in our study.

The majority of the pediatric patients were found within the age group of 5 to 9 years. The result of this study revealed that age is weakly and negatively associated with serum levels of potassium (*r* = − 0.043, *p* > 0.05 and *r* = − 0.020, *p* > 0.05), calcium (*r* = − 0.15, *p* > 0.05 and *r* = − 0.14, *p* > 0.05), uric acid (*r* = − 0.11, *p* > 0.05 and r = − 0.04, *p* > 0.05), LDH (*r* = − 0.07, *p* > 0.05 and *r* = − 0.06, *p* > 0.05), and total WBC count (*r* = − 0.05, *p* > 0.05 and *r* = − 0.08, *p* > 0.05) respectively before and after treatment, but only serum creatinine level before treatment was statistically significant. At the same time, age was weakly and positively associated with serum BUN (*r* = 0.14, *p* > 0.05 and *r* = 0.20, *p* > 0.05) and creatinine (*r* = 0.27, *p* < 0.05, and *r* = 0.15, *p* > 0.05) respectively before and after treatment. There is a natural tendency that as age increases the muscle mass tends to increase, and also during cancer there is increased muscle wasting leading to a proportioned increase in creatinine level as a catabolic product of amino acids.

Results showed that gender did not have statistically significant association with the occurrence of TLS (X^2^ = 1.378, *p* > 0.240). This finding does not agree with a previous study which reported that males are more susceptible to TLS [15]. This requires a further elaborated study with a larger sample size and gender balance. Residence (X^2^ = 0.105, *p* > 0.05) and family income (X^2^ = 1.625, *p* > 0.202) did not show statistically significant association with the occurrence of TLS in the study participants. But in previous studies, children from low and middle income countries were shown to be at a greater risk of developing TLS [16, 17]. In our case almost all the study participants were in low income stratification hence there was no significant variation within the group with respect to TLS occurrence. Nearly 90% of the patients recovered from TLS during the follow up period. There were 2 (11.1%) death incidences as a consequence of TLS complication. There is a variation in death incidence report due to TLS across different research reports [10, 11]. The variation in the incidence of death report may be due to the difference in the treatment protocols and the management, the variation in the study population and the nature of the underlying malignancies. A recent review can be referred for more information on TLS [18].

## Conclusions

In this study TLS occurred irrespective of gender, residence and family income. This suggests that every child with cancer is at risk of developing TLS. Early identification of patients at risk and prevention is of crucial importance. Spontaneous TLS was more prevalent than the drug induced one. This may indicate that patients do not come to health facility early before complication and have advanced malignancy with high tumor burden. The biochemical parameters studied in this research and in other studies have shown strong association with the occurrence of TLS. Thus, these parameters shall be considered in TLS prediction, diagnosis and prognosis. TLS incidence was high irrespective of intensive prophylactic managements (i.e., hydration and allopurinol). A better management strategy shall be adapted in the clinics and valid and efficient diagnostic tests shall be used for better diagnosis and prognosis of cancer and TLS.
